# Genome-wide scan for potential CD4+ T-cell vaccine candidates in *Candida auris* by exploiting reverse vaccinology and evolutionary information

**DOI:** 10.3389/fmed.2022.1008527

**Published:** 2022-11-03

**Authors:** Shishir K. Gupta, Özge Osmanoglu, Rashmi Minocha, Sourish Reddy Bandi, Elena Bencurova, Mugdha Srivastava, Thomas Dandekar

**Affiliations:** ^1^Department of Bioinformatics, Biocenter, Functional Genomics and Systems Biology Group, University of Würzburg, Würzburg, Germany; ^2^Evolutionary Genomics Group, Center for Computational and Theoretical Biology, University of Würzburg, Würzburg, Germany; ^3^Department of Biochemistry, All India Institute of Medical Sciences, New Delhi, India; ^4^Institute of Experimental Biomedicine, University Hospital Würzburg, Würzburg, Germany; ^5^Core Unit Systems Medicine, University of Würzburg, Würzburg, Germany; ^6^BioComputing Unit, European Molecular Biology Laboratory (EMBL), Heidelberg, Germany

**Keywords:** *Candida auris*, T-cell epitope, epitope prediction, positive selection, evolution, immune-informatics

## Abstract

*Candida auris* is a globally emerging fungal pathogen responsible for causing nosocomial outbreaks in healthcare associated settings. It is known to cause infection in all age groups and exhibits multi-drug resistance with high potential for horizontal transmission. Because of this reason combined with limited therapeutic choices available, *C. auris* infection has been acknowledged as a potential risk for causing a future pandemic, and thus seeking a promising strategy for its treatment is imperative. Here, we combined evolutionary information with reverse vaccinology approach to identify novel epitopes for vaccine design that could elicit CD4+ T-cell responses against *C. auris.* To this end, we extensively scanned the family of proteins encoded by *C. auris* genome. In addition, a pathogen may acquire substitutions in epitopes over a period of time which could cause its escape from the immune response thus rendering the vaccine ineffective. To lower this possibility in our design, we eliminated all rapidly evolving genes of *C. auris* with positive selection. We further employed highly conserved regions of multiple *C. auris* strains and identified two immunogenic and antigenic T-cell epitopes that could generate the most effective immune response against *C. auris.* The antigenicity scores of our predicted vaccine candidates were calculated as 0.85 and 1.88 where 0.5 is the threshold for prediction of fungal antigenic sequences. Based on our results, we conclude that our vaccine candidates have the potential to be successfully employed for the treatment of *C. auris* infection. However, *in vivo* experiments are imperative to further demonstrate the efficacy of our design.

## Introduction

In recent years, life-threatening fungal diseases have increased, and new infections have emerged. Selection pressure of climate change has significantly contributed to the emergence of *Candida auris* as a pathogen (1). *C. auris*, a multidrug-resistant ascomycete, was first isolated in 2009 in Japan. Up to date, it has been detected in 32 countries over six continents (2). *C. auris* infects various tissues and organs, including central nervous system (3), cardiovascular system (4), respiratory tract (5), bones and joints (6), and possesses the potential to cause nosocomial infections (7). The diagnosis of *C. auris* is difficult due to non-availability of specific laboratory techniques which could rapidly and accurately detect it in clinical samples. Furthermore, treatment of infections caused by *C. auris* poses a real challenge due to its high multidrug-resistivity pattern. The reduced susceptibility to conventional antifungal drugs such as azoles and amphotericin B leads to high mortality rate, rising up to 60% (8, 9). Furthermore, *C. auris* can persist on abiotic surfaces such as healthcare instruments for several weeks, which can facilitate prolonged pathogen persistence and high transmissibility (10). This also explains why transmission of *C. auris* to hosts frequently occurs in hospitals. Its persistence in the environment is a trait that differentiates it from other *Candida* species.

Development of anti-fungal vaccines which can trigger the host immune response to generate immunological memory against fungi and their spores remains challenging. Nevertheless, three approaches of immunization are commonly used in development of anti-fungal vaccines; (i) vaccination with live-attenuated strains, which can be hazardous due to possible disease development in immunocompromised patients (11), (ii) immunization with recombinant proteins containing the immunogenic sequences (epitopes), or (iii) immunization with polysaccharides, which are present only in fungal cell wall (12).

To date, there is no human vaccine approved against *C. auris* infections, however, several research groups have made significant progress in its development. Currently, most studies are focused on the Als3, a member of agglutinin-like sequence (Als) family of proteins with adhesive and invasive properties (13). Researchers have proposed Als3 as a promising therapeutic target for *Candida* albicans vaccine development (14–17). In a recent study, Singh et al. (18) identified three adhesin/invasin proteins in *C. auris*, that shared sequence and structural homology to Als3 protein of *C. albicans*. The NDV-3A vaccine proposed by Singh et al. (18) which was based on the N-terminus of Asl3-protein sequence significantly blocked biofilm production ability of *C. auris in vitro.* These authors also found that when combined with the antifungal drug micafungin, the NDV-3A vaccine augmented the protective efficacy of this drug against *C. auris* infection in neutropenia mice. It further induced regulatory CD4+ T helper (TH) cells in infected mice, which comprised of Th1, Th2, and Th17 subcellular population (18). In another study, immunizing mice with rAls3p-N vaccine induced T-cell mediated protection in *C. albicans*, which further signifies the crucial role of CD4+ T lymphocytes as well as associated cytokines such as IFN-γ in acquired immune response against the pathogen (19, 20).

The objective of the current study was to identify novel putative CD4+ T-cell epitopes with vaccine potential against *C. auris* infection by harnessing the evolutionary information combined with reverse vaccinology approach. To this end, we extensively scanned the family of proteins coded by *C. auris* genome and identified the potential vaccine candidates. In search of epitope-based vaccine candidates, one could expect that the pathogen may acquire substitutions in epitopes targeted by immune memory over the years. For instance, hemagglutinin protein of influenza A evolves under strong selection from antibodies (21). To avoid such possibilities, we computed non-synonymous and synonymous distances and then tested for sites with statistical evidence where the accumulation of non-synonymous substitutions exceeds that of the synonymous substitutions. We thus discarded all the rapidly evolved genes of *C. auris* with positively selected sites. We further used the highly conserved regions of protein sequence alignment of multiple *C. auris* strains, so as to identify major candidates for designing the new potential vaccine. This subsequently decreased the possibility of epitopes to rapidly evolve and escape the immune recognition. Moreover, using the stringent *in silico* analysis, we finally identified two conserved immunogenic and antigenic CD4+ T-cell epitopes that could be used for efficient immune memory generation against *C. auris*.

## Materials and methods

### Genome sequences and quality control

The protein coding genes and translated transcriptomes of five *C. auris* strains (*C. auris* 6,684 from India, *C. auris* B8441 from Pakistan, *C. auris* B11220 from Japan, *C. auris* B11221 from South Africa, and *C. auris* B11243 from Venezuela) were retrieved from NCBI GenBank release 231.0 (22). The annotation completeness of the genome assemblies was accessed with BUSCO v3 (23) using Fungi odb9 database that contains single-copy orthologs (SCOs) selected from OrthoDB v9 (24, 25). Supplementary Table 1 lists the version and details of the genomic assembly.

### Identification of orthologs

Clustering of orthologous genes was performed using Orthofinder v2.3.3 (26) with Diamond v0.9.24.125 (27) (under default settings) based on translated proteomes of selected five strains. The orthologous clusters that did not contain orthologous genes from all the selected five strains were discarded to only keep genes conserved in all strains. Among the genes in each conserved orthologous clusters, genes that belong to *C. auris* B11220 (4,860 genes in total) were used as reference for further analysis due to higher quality and annotation completeness of its assembly.

### Filtering of candidate genes

Conserved *C. auris* B11220 genes were filtered to eliminate the unlikely vaccine candidates. Table 1 list the tools used for filtering. The first round of elimination was performed based on their secretion or cellular localization. Secretion of the proteins was predicted by checking whether they have a signal peptide or a glycosylphosphatidylinositol (GPI)-anchor or a transmembrane (TM) domain. Signal peptides were identified by using TargetP (28), SignalP (29), Phobius (30), and FunsecKB2 (31). GPI-anchor were identified using PredGPI (32). To identify TM domain, TMHMM (33) and Phobius (30) were used.

**TABLE 1:** Web-servers and databases used for sequence filtering.

Name	Description	Method	References
DeepLoc 2.0	Eukaryotic protein subcellular localization	Machine learning-deep neural networks	97
EffectorP 2.0	Fungal effector proteins	Machine learning	37
PredGPI	GPI-anchor prediction	Support vector machine (SVM) and hidden markov model (HMM)	32
TargetP	Eukaryotic protein subcellular localization	Neural network	98
FungalRV	Fungal adhesin prediction	SVM	38
Phobius	Transmembrane topology and signal peptide prediction	Hidden markov model (HMM	99
SignalP 6.0	Signal peptide prediction	Deep neural network	100
TMHMM 2.0	Transmembrane helices prediction	Hidden markov model	33
FAAPred	Fungal adhesins and adhesin-like protein prediction	Support vector machine (SVM)	39
FunSecKB2	A fungal protein subcellular location knowledgebase		101
ScanProsite	PROSITE signature detection		34

Next, proteins with any of the features predicting their sorting *via* classical secretion pathway were further filtered based on the presence of endoplasmic reticulum (ER) retention signal that may restrict their secretion to extracellular space or cell membrane. This step of filtering through the detection of PROSITE pattern PS00014 was performed by ScanProsite (34). Last step of the first round of elimination was done by sorting the proteins by the presence of a signal peptide or a TM domain or a GPI anchor. Then the cellular localization of the proteins was assessed by Deeploc (35). Accordingly, the proteins with a signal peptide were filtered further by their cellular localization being cell membrane, while the proteins with TM domain or GPI anchor were reduced to the ones with extracellular localization.

Second round of filtering was based on fungal effectors and adhesins which possess a higher probability of being a vaccine target (36). The filtering was performed using prediction tools EffectorP (37), FungalRV (38), and FaaPred (39) on both sets of proteins that were subject to classical secretion pathway either to the cell membrane or extracellular space.

### Positive selection analysis

Proteins identified from the previous step were aligned with their orthologs from other *C. auris* strains under study. T-Coffee (40) was used for the multiple sequence alignment (MSA) which combines the output of different aligners to enhance the MSA accuracy. The aligned amino acid sequences together with the corresponding nucleotide sequences of each ortholog group were converted into nucleotide alignments at the codon level using the program PAL2NAL (41). Since removal of unreliable regions increases the power to detect positive selection, we used stringent Gblocks filtering (type = codons; minimum length of a block = 4; no gaps allowed) to remove gap-rich regions from the alignments (42). We used site model implemented in codeml program from the PAML 4.2b package (43) to detect the sites in the alignment under positive selection. The Bayes empirical Bayes approach was employed to estimate the probabilities of positive selection for specific codons under the likelihood framework (44). FEL (45) and MEME (46) were used to evaluate for positive selection among filtered genes. The cut off parameter was set to *p* < 0.01. If the positive selection was detected by any of mentioned three programs, the genes were removed for the subsequent analysis.

### Highly conserved regions of ortholog clusters

Conservation of amino acid residues in the aligned pathogen sequences was estimated by Shannon entropy function (47) using the Protein Variability Server.^1^ We selected the Shannon entropy variability threshold of 1.0 to extract the highly conserved consensus subsequences of length >9 mers. The Shannon entropy (S) for every position in the sequence alignment was calculated as


$$S=-\sum_{i=0}^{N}F\,i\,{\text{log}}_{2}\,F\,i$$

where *Fi* is the fraction of residues of amino acid type *i*, and *N* is the number of amino acid types (48).

### CD4+ T-cell binding peptide prediction

The peptides that trigger major histocompatibility complex (MHC) class-II responses are often longer than class-I peptides. These MHC binders are <13–15 amino acids longer with a core sequence of about 9 mers, usually three anchor residues, and their ends extend beyond the peptide-binding groove (49, 50). The MHC class-II binding 13–15 mer peptides for alleles were predicted using Immune Epitope Database (IEDB) (51) recommended prediction method and on full HLA reference set with percentile rank.

### Host similarities and antigenicity of MHC class-II binders

To assess the similarity of the predicted MHC class-II binders with human sequences, Blast similarity search (52) was performed. The Blast parameters were tailored appropriately for this analysis, in accordance with the short size of the peptides (such as matrix PAM30; word-size 2). We first screen the binders against human assembly from ENSEMBL (assembly version GRCh38.p13) and then against NCBI non-redundant human sequence database. Highly similar sequences were discarded from further evaluation. We further performed Blast analysis against a customized sequence database of experimentally verified autoimmune class-II epitopes downloaded from IEDB database (51) to avoid the induction of potential autoimmune reactions. Only the sequence with less than 35% sequence identity over 80% query coverage were evaluated for the antigenicity using antigenicity prediction server^2^ and Vaxijen server (53). VaxiJen is based on auto cross covariance (ACC) transformation (53) of protein sequences into uniform vectors of principal amino acid properties, with a threshold value 0.5 for prediction of fungal antigenic sequences. Only consensus predictions from the antigenicity prediction server were considered.

### Population coverage

Before population coverage analysis the filtered epitopes binding affinity for HLA class II alleles was accessed using MHC-II prediction tool at IEDB using the neural align method 2.3 (NetMHCII 2.3) and epitope length 11–15 amino acids. Resulting alleles were sorted according to their IC50 (inhibition concentration 50) values and only alleles with IC50 equal to or less than 500 nM were selected for further analysis. For the calculation of the population coverage, selected alleles were examined by IEDB Population coverage tool. This analysis was performed for 16 different geographical regions and global population.

## Results

With the aim to identify novel putative CD4+ T-cell epitopes against *C. auris* infection, a pipeline was developed including several steps based on orthology, cellular localization, positive selection, MSAs, T-cell binding potential, antigenicity, and population coverage.

### *Candida auris* B11220 as representative of five strains

**FIGURE 1:**
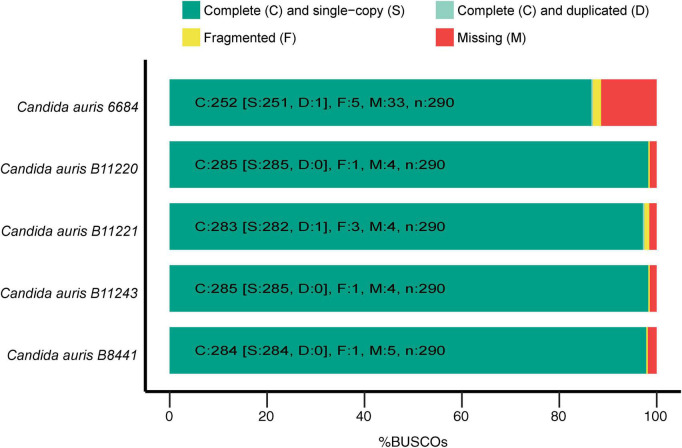
Genome statistics. The graph depicts the genome completeness of the investigated *Candida auris* strains as determined by benchmarking universal single-copy orthologs (BUSCO), with BUSCOs for Fungi (odb9) serving as the background.

Genome assemblies of *C. auris* strains originating from different geographical locations were used for the first step of assessing possible common genes between different strains to be adopted as potential vaccine targets. All the used strains had almost similar sizes in the range of 12.1–12.7 Mb with GC-content of ∼45%. These genomes were originally assembled at scaffold level with scaffold N50 ranging between 60 and 2400 × 10^3^ bp (Supplementary Table 1). Next, the Benchmarking Universal Single-Copy Orthologs (BUSCO) (23) software was used for assessment of the quality and completeness of assembly and annotation of the used *C. auris* strains. Employing hidden Markov model (HMM) (54) profiles for a match between given proteome and the OrthoDBv9 (24, 25) SCO dataset for fungi, BUSCO determines the fraction of complete, fragmented, and missing genes for each genome. In other words, it assesses the quality of the genomic data based on expected gene content, which is determined/guided by the fungal SCO dataset. As mentioned above, low quality caused by technical issues in genome assemblies can be detected by metrics like N50 for contigs or scaffolds. However, quality of the genome resource that can be affected by, for instance, contamination, is also influential for the comparative downstream analyses. Therefore, to detect any quality issues with the genomic resource, BUSCO uses an expected gene set of SCO as marker genes for genome/assembly completeness (55). Genes that were marked as complete were further classified in single-copy and duplicated and quantification of all fractions was thereby assessed by BUSCO. The *C. auris* 6,684 strain assembly showed the lowest completeness level with 33 missing genes and five fragmented genes, whereas the highest quality of assembly and annotation was observed for *C. auris* strains B11220 and B11243 with 285 out of 290 complete genes (Figure 1). We identified a total number of 5,331 orthologous groups (OG) among five strains of *C. auris.* In our analysis, 4,752 out of 5,331 OGs were found to encompass all five strains and 4,616 OG were found to be single copy orthologs clusters. B12221 strain of *C. auris* was determined to have the highest number of duplication events with 49 duplications and the highest number of genes with 118 genes to have more than one copy. *C. auris* 6,684 strain was found to be missing in 405 OG, missing in highest number of OG among five strains.

**FIGURE 2:**
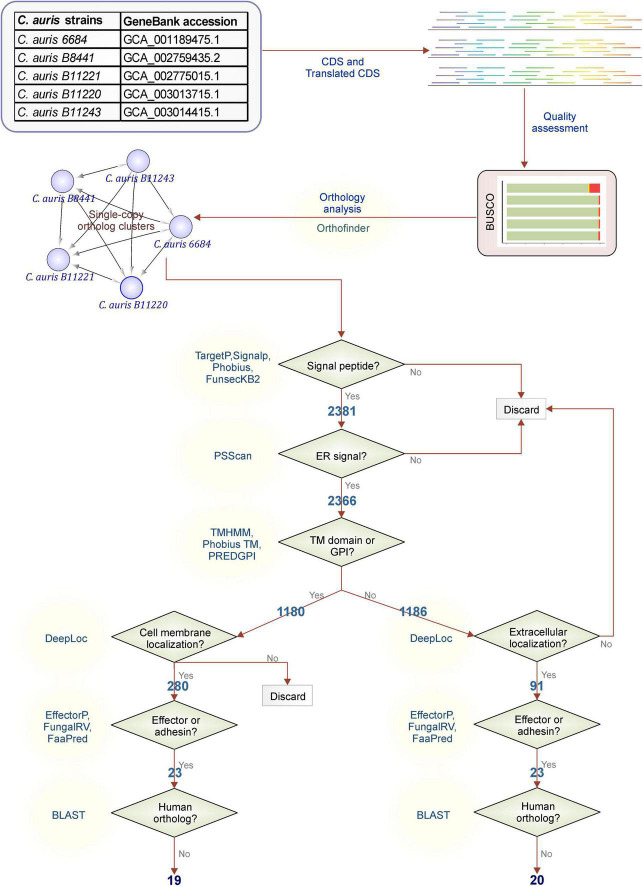
Filtering workflow. Steps used for *C. auris* protein filtering adapted from Vivek-Ananth et al. (79) The *C. auris* proteins with orthologs in all strains were filtered to obtain membrane proteins or extracellular secreted proteins. Secretion to the extracellular space or to the membrane was determined by existence of a signal peptide or glycosylphosphatidylinositol (GPI) anchor or a transmembrane (TM) domain. The proteins with a endoplasmic reticulum (ER) signal peptides were filtered out to ensure the localization of the protein to the membrane or extracellular space. Further steps were taken to predict the effector or adhesins and to filter out proteins that may invoke cross-reactivity.

Based on our orthology analysis, assembly quality, and genome completeness, *C. auris* strain B11220 was selected for further analysis and steps of filtering of orthologous gene clusters were followed as shown in Figure 2.

### Filtering pipeline identifies candidate genes with possible extracellular or membrane localization

First, filtering was performed for identification of genes with possible signaling routes by controlling following features: the existence of a signal peptide with TargetP (28), SignalP (29), Phobius (30), and FunsecKB2 (31), the existence of a GPI anchor with PredGPI (32), and the existence of a TM domain with TMHMM (33) and Phobius (30). Elimination of genes with no probability of being signaled into or through membranes led to a reduction to 2,381 genes that were further eliminated in case of the presence of ER retention signal. Further classification of these signaling proteins was performed through assessment of location by testing the presence of a TM domain or a GPI-anchor (30, 32, 33). Almost half (1,180) of 2,366 possibly signaled proteins were found to possess TM domains or a GPI-anchor. These 1,180 proteins detected here were then further filtered by their location determined by DeepLoc (35). A total of 280 proteins were detected with no cell membrane, and possibly secreted *via* the classical secretion pathway. On the other hand, the other half without TM domains or a GPI-anchor (1,186 proteins) were filtered down to 91 proteins by their location being extracellular. Next step identified 23 proteins for each class as effector or adhesion proteins that may have a higher probability to be a good vaccine target. Out of 23 membrane effector or adhesion proteins, 4 were filtered due to high similarity to human proteins while for extracellular effector or adhesion proteins only 3 were eliminated in this step. This last step ended in 19 membrane and 20 extracellular proteins with low or no similarity to human proteins that reduces the risk of cross-reactivity with host’s self-proteins.

Next, the 39 proteins clustered in 38 orthogroups were checked for signs of positive selection. Out of 38 gene alignments, 5 were identified by our pipeline with sites under positive selection (Supplementary Dataset 1). PAML (43) identified sites under selection in orthogroups OG0003318 and OG0003714. Selection in OG0000005 was identified by both FEL (45) and MEME (46). In addition, MEME also identified selection in OG0000033 and OG0004411. These genes were removed for the subsequent analysis. PAML identifies the sites that could be under continuous changes while MEME identifies the episodic positive selection which implies that site changes are kept in clade to provide the advantage in the new environment. As a consequence of selection pressure from the host environment, *C. auris* like other pathogens can undergo evolutionary changes for enhanced survival.

### Conserved regions reveal immunogenic and antigenic peptides

For the proteins coded from the genes with no sign of positive selection, we then focused on highly conserved regions of their MSA. This is particularly important to increase the coverage of vaccine candidates as the regions of low Shannon entropy would theoretically remain conserved even upon adding the new *C. auris* strains in the MSA. Therefore, we selected the candidate proteins from conserved regions using Shannon entropy as previously used (56–58). Shannon entropy reflects the degree of variability of protein sequence fragments and supports their evolutionary stability inferences. Stable peptides are characterized by low entropy and an entropy value of 2.0 indicates conserved fragments. With increasing variability of a site, entropy increases and is influenced by both the number of variants at that site and their respective frequency. The estimated average conservancy less than 1.0 postulated that the proposed epitopes would be highly conserved among used *C. auris* strains. We used these conserved fragments originated from 38 alignments and in total identified 7,149 CD4+ T-cell binders.

If the predicted peptides share a higher identity with host genomic regions, they can be imitated as self-molecules. The ability to prevent immunological responses against self-antigens is advantageous which would liberate the vaccine from the risk of inducing autoimmunity. Therefore, to avoid the possibility of causing autoimmunity because of the homology of predicted MHC class-II binders with humans, we performed the blast similarity search analysis (see Materials and methods). For searching against IEDB auto IEDB we used stringent cutoff and only selected the MHC-binders that have less than 35% identity over 80% sequence coverage. This removed >99% of predicted MHC-binders and only 6 MHC-binders could cross this threshold (Supplementary Table 2). Such reduction was not surprising because of the close evolutionary distance between the eukaryotic pathogen *C. auris* and humans (1,105 Mya) compared to bacterial (such as *Mycobacterium tuberculosis*; 4,290 Mya) pathogens as estimated with TimeTree (59).

**FIGURE 3:**
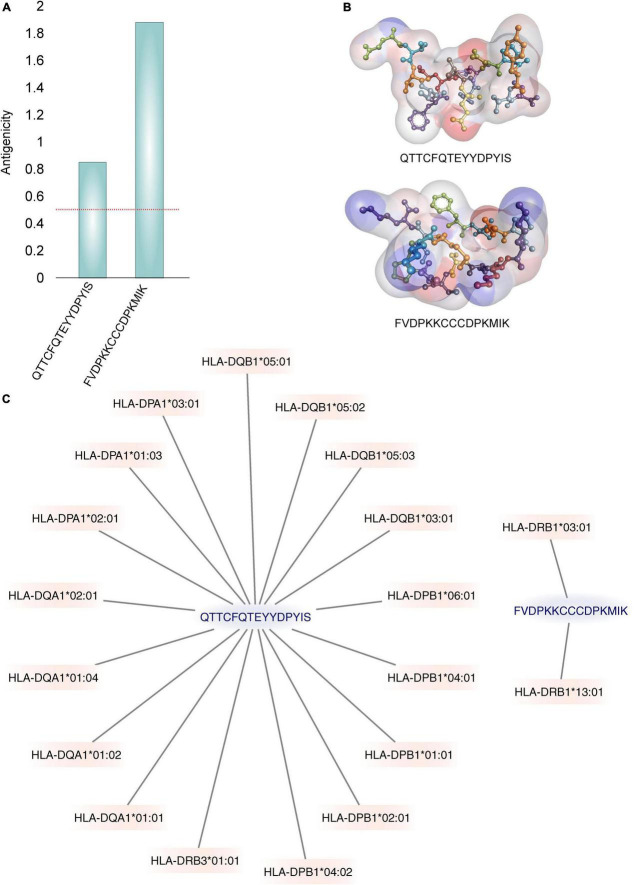
The best two identified epitopes. **(A)** The Vaxijen server’s calculated antigenic potential (96) is displayed. A horizontal red line indicates the antigenicity threshold. **(B)** Illustrations of the 3D structures of epitopes. The residues are shown under a soft surface and are colored by atom charges. **(C)** Graphs are used to show how binding with MHC-II alleles is displayed. Only the alleles with binding IC50 values ≤500 nM are displayed.

Furthermore, only the ability to bind to the MHC receptor does not guarantee that the binder peptide is antigenic. Out of six, only two predicted MHC class-II binders were found to be antigenic. VaxiJen antigenicity score of QTTCFQTEYYDPYIS and FVDPKKCCCDPKMIK was 0.85 and 1.88, respectively with probable fungal antigen annotation at 0.5 threshold (Figure 3A). The 3D structure of these epitopes is shown in Figure 3B.

### High population coverage is required for the recognition of epitopes

**FIGURE 4:**
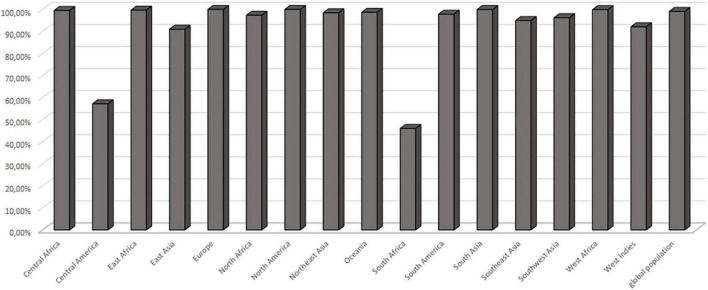
Population coverage. Cumulative population coverage for both predicted epitopes QTTCFQTEYYDPYIS and FVDPKKCCCDPKMIK.

The MHC class II molecules are known to be highly polymorphic and are critically involved in recognition and defense against pathogens. The distribution of MHC alleles differs among various ethnic groups worldwide. Therefore, a peptide that acts as a T-cell epitope in majority of a population with a particular MHC alleles distribution may not work in a population with a different MHC alleles distribution. Thus, allelic distribution is crucial also for the adaptation of the population to environmental changes and it various among different geographical regions (60). Both identified epitopes were therefore submitted to IEDB server as described in methodology section. Only MHC class II binders with strong to intermediate binding affinity (IC50 less or equal to 500 nM) were selected to calculate population coverage (Figure 3C and Table 2). The analysis of both epitopes revealed high coverage for the global population (99.09%). Therefore, we assume that the identified epitopes can be recognized by sufficient HLA alleles and cover most of the world’s population. However, we noted significantly lower binding abilities in Central American (57.2%) and South African (45.98%) regions (Figure 4), thus only approximately half of the population can recognize selected epitopes. The low binding ability is due to the small number of the epitopes (only 2 epitopes were examined) and low IC50 value. The IC50 value ≤500 was selected to obtain more accurate results when even lower load of fungi can be recognized by immune cells, which is crucial mainly for the immunocompromised patients. However, selecting more benevolent values, the number of the binding alleles would increase and thus also the probability for the better coverage in Central American and South African population, where candidemia caused by *C. auris* accounts for 10% of all cases (61).

**TABLE 2:** List of selected alleles used for the population coverage analysis.

Epitope	Allele	IC50	Percentile rank	Allele locus	Prediction method
QTTCFQTEYYDPYIS	HLA-DRB3*01:01	205.6	6.2	HLA-DR	NN-align 2.3
QTTCFQTEYYDPYIS	HLA-DQA1*01:01/DQB1*05:01	17.2	0.02	HLA-DQ	NN-align 2.3
QTTCFQTEYYDPYIS	HLA-DQA1*01:02/DQB1*05:02	278.5	1.8	HLA-DQ	NN-align 2.3
QTTCFQTEYYDPYIS	HLA-DQA1*01:04/DQB1*05:03	290.9	0.31	HLA-DQ	NN-align 2.3
QTTCFQTEYYDPYIS	HLA-DQA1*02:01/DQB1*03:01	359.7	34	HLA-DQ	NN-align 2.3
QTTCFQTEYYDPYIS	HLA-DPA1*01:03/DPB1*06:01	17.40	2.2	HLA-DP	NN-align 2.3
QTTCFQTEYYDPYIS	HLA-DPA1*01:03/DPB1*04:01	35.10	0.51	HLA-DP	NN-align 2.3
QTTCFQTEYYDPYIS	HLA-DPA1*02:01/DPB1*01:01	198.60	2	HLA-DP	NN-align 2.3
QTTCFQTEYYDPYIS	HLA-DPA1*01:03/DPB1*02:01	256.00	6.9	HLA-DP	NN-align 2.3
QTTCFQTEYYDPYIS	HLA-DPA1*03:01/DPB1*04:02	370.4	6.5	HLA-DP	NN-align 2.3
FVDPKKCCCDPKMIK	HLA-DRB1*13:01	368.1	50	HLA-DR	NN-align 2.3
FVDPKKCCCDPKMIK	HLA-DRB1*03:01	430.5	6.3	HLA-DR	NN-align 2.3

## Discussion

The best strategy to control an infectious disease in a given population and one of the most efficient, quickest, and affordable ways to promote public health is through vaccination. Given the fact that *C. auris* can be resistant to nearly all available antifungal medications, the most effective way to fight this evolving and fatal pathogen is potentially by using a vaccine targeted against it. Notably, epitope-based vaccines are known to generate a stronger immune response against a pathogen as compared to the whole protein vaccines (62). Previously, vaccine development was solely dependent on experimental techniques which required extensive resources and time. However, with recent advancements in the fields of bioinformatics and reverse vaccinology, as pioneered by the work of Rino Rappouli, (63), we have efficient *in silico* approaches to screen the genome for best epitopes which can thus be employed to design novel vaccines in a cost and time effective manner. These approaches have been already successfully employed by many researchers for identifying potent vaccine candidates (56, 64–67), which further endorses the potential of *in silico* approaches in vaccinology. Reverse vaccinology is now especially poised to produce more vaccines after the promising discovery of the first accepted multicomponent meningococcal serogroup B (MenB) vaccine (4CMenB; Bexsero^®^) which was proposed from computational approaches and later received huge success in laboratory experiments (63, 68).

Th1 cells (a subtype of CD4+ TH cells) are known to play a central role in providing immunity against fungi and effective fungal vaccines. They also induce generation of pro-inflammatory signature cytokines IFN-γ and TNF-α and play a role in production of opsonizing antibodies which promotes enhanced phagocytosis at the sites of infection (69). In addition, emerging evidence states that Th17 cells (another subtype of CD4+ TH cells) have also been implicated in the generation of specialized immune response against fungal infection and usually a balance between the Th1 and Th17 associated responses is desired (69, 70). In memory pool of humans, anti-fungal Th17 memory cells have been found and they have also been known to play a role in induced vaccine protection in mice (71). Furthermore, Th17 cells act majorly at mucosal surfaces and thus, Th17 cells inducing vaccines are adequate to protect against deadly lung infection caused by *Blastomyces dermatitidis* in mice and also against three major systemic mycoses in North America (72). Altogether, it is highly suggested to target Th17 induction in the potential vaccine designs against systemic fungal infections (73).

We have previously developed and used different strategies to increase the ability of computer-aided vaccine designing approaches to identify vaccine candidates such as (1) epitope selection from conserved regions (74), (2) profile similarity method to analyze biased-ness of predicted epitopes toward profiles of experimentally validated epitopes (74), (3) introducing cleavage sites for targeted cleavage of multi-epitopic vaccine (56, 75), (4) B-cell epitope prediction by docking (74), (5) structure-based epitope identification (57), (6) CpG optimization (76), (7) sequence homology search against host (77), (8) adding adjuvant such as IL-12 (78), and (9) population coverage analysis (58, 77). In this study, we integrated the evolutionary analysis with our reverse vaccinology, i.e., bioinformatical genome and epitope search pipeline (74, 77) to identify vaccine candidates. The approach we used can be particularly helpful for designing vaccine against any emerging pathogens. Here we used this for epitope identification for potential vaccine against *C. auris*.

Currently, only the NDV-3A vaccine has shown potential so far in immunizing mice against *C. auris* infection (18). We extensively scanned the whole *C. auris* genome coded candidate proteins for their potential as vaccine candidates. We focused on secreted proteins and cell membrane proteins as they are particularly important for fungal host-pathogen interactions. During pathogenesis, these proteins play a role in the interaction with the host immune cells. Moreover, they are good candidates for targeting because of easier access and low expected-resistance mechanisms (79). Therefore, we followed the workflow in Figure 2 [adapted from Vivek-Ananth et al. (79)] to filter *C. auris* proteins based on existence of a signal peptide, a GPI anchor or a TM domain. The filtered proteins were then classified in two separate groups of secreted and membrane proteins.

Five major clades of *C. auris* have been identified up to date with tens to hundreds of thousands of single nucleotide polymorphisms, which can be potentially linked to various infection strategies and outbreaks (80, 81). It had been shown that highly related isolates play important role in local and ongoing transmission, however, undergone clonal expansion was detected in each clade (82). Moreover, as fungus has evolved as a pathogen under strong selection pressure imposed by climate change, it is important to recognize the rapidly evolved genes of *C. auris* (83). This knowledge is critical not only to understand the transmission dynamics, but also to design the suitable vaccine. In comparison of other genes, rapidly evolved genes have higher possibility to mutate to facilitate the pathogen survival in critical environment. We identified the positive selection signals and removed the proteins coded by such genes. Studies have shown that mutations in viral epitopes can promote hindrance in viral recognition by CD4+ and CD8+ T-cell receptors leading to escape of viruses from immune surveillance, thus escalating pathologic conditions (84). For development of a successful *C. auris* vaccine, it is crucial to have a memory pool of T-cells which can recognize *C. auris* and provide immunity despite its continuous evolution. To maintain high affinity, T-cell memory pool epitopes which are highly conserved and resistant to mutations should be considered. Thus, we focused on the conserved regions of MSAs after elimination of ortholog clusters with signs of positive selection.

Finally, we determined candidates from the conserved regions that are highly immunogenic, antigenic, and that show no significant similarity with host proteins. Furthermore, we also analyzed if the identified MHC class-II epitopes showed any similarity to other pathogenic organisms including fungi (taxid4751), bacteria (taxid2) and parasites from clade of *Protostomia* (taxid33317) that include parasitic worms *Nematoda* and *Platyhelminthes* and other parasites like *Trypanosomatida* (txid2704949) and *Plasmodium* (txid5820). We performed our Blast search with the parameters adjusted for short sequence length (see Host similarities and antigenicity of MHC class-II binders). Our search against bacteria and chosen parasite sequences showed no similar hits to both epitopes (*e*-value < 0.01). On the other hand, we have found seven fungal hits for epitope 1 with the *e*-value lower than 0.01. All hits showed 85.71% identity and 93.3% query coverage (Supplementary Figure 1). Six of seven hits are from *Hanseniaspora* species, a yeast genus while 1 is from *Brettanomyces*, a non-spore forming yeast genus. Thus, we also performed more specific Blast search for more possible hits in yeast first against the *Saccharomycetes* (txid4891) class and then directly with the *Candida* genus (txid5475, excluding *C. auris* from the search) of the same class. We found no hits with *e*-value < 0.01 in other *Candida* species for any of the epitopes. However, we found that 42 yeast proteins show similarity to epitope 1 (*e*-values < 0.01) and further investigated these hits. Top seven hits showed query coverage higher than 90% and identity score higher than 85%. When we compared these hits to the previous fungal proteins, we found a 100% overlap. The 17 of the remaining hits also had identity score over 78% and query coverage over 85% for epitope 1. Finally, the 18 remaining hits only showed identity scores higher than 70% and query coverage higher than 60% and thus were not determined to be similar to epitope 1.

In our work, we used 5 *C. auris* strains spanning 4 clades namely *C. auris* 6,684 Clade I from India, *C. auris* B8441 Clade I from Pakistan, *C. auris* B11220 Clade II from Japan, *C. auris* B11221 Clade III from South Africa, and *C. auris* B11243 Clade IV from Venezuela. For further analysis to determine the clade coverage of our epitopes we also performed a protein sequence comparison against *Candida auris* (taxid:498019). This is especially important, since our analysis did not include the Iran strain IFRC2087, belonging to the recently identified clade V. We found 5 hits that showed 100% identity and coverage to epitope 1 from strains 6,684 (India, Clade I), and JCM 15448 (Japan, Clade II), B11221 (South Africa, Clade III), B11243 (Venezuela, Clade IV), and IFRC2087 (Iran, Clade V). For epitope 2, we also found 5 hits from all clades, respectively from strains 6,684 (India, Clade I), B11220 (Japan, Clade II), B11221 (South Africa, Clade III), B11243 (Venezuela, Clade IV), and IFRC2087 (Iran, Clade V) (Supplementary Table 3). Furthermore, since many isolates from different strains only have their genomes sequenced and no protein annotation yet, we have also performed a blast search in the nucleotide database with tblastn algorithm. Our analysis revealed 43 hits for each epitope (with 100% coverage and identity). Moreover, we found these hits to be from the same strains for both epitopes. Among these strains, 32 belong to clade I (28 from Lebanon: Beirut, 1 from China: Beijing, 1 from India, 1 from Italy: Genoa, and 1 from United Arab Emirates), 5 belong to clade II (1 from each Canada: Alberta, China: Shenyang, Japan, South Korea, and USA: New York), 4 belong to clade III (1 from each Canada: Quebec, South Africa, USA: California, USA: Indiana), 2 belong to clade IV (Colombia: Cartagena, and Venezuela) and 1 belongs to clade V from Iran (Supplementary Figure 2).

Taking all these results and comparison into account, we further decided to combine these two epitopes using the linker KFERQ (56, 85) so as to generate a more potent immune response from our vaccine construct. We thus performed *in silico* simulations for possible immune responses using the C-IMMSIM server (86, 87). Two injections were given with 4 weeks interval and immune response over a 6 months-long period was predicted. We have used the alleles that we determined to cover larger portion of the global population (namely HLA-DRB1*13:01, HLA-DRB1*03:01, and HLA-DRB3*01:01 since only these three were included in the database). Predicted response for all three combinations of the HLA alleles showed high innate response after the first injection and even higher IgM counts after the second injection. This demonstrates long-term immune response which is still retained upto 6 months (Supplementary Figure 3). We also observed an increase in the memory CD4+ T-cells’ number to as high as 1,600 cells per mm^3^ after the second injection and still a population of 100 cells per mm^3^ even after 6 months (Supplementary Figure 4).

Since 2019, SARS-CoV-2 belongs to one of the most prevalent viral pathogens worldwide. It is well documented that COVID-19 patients often suffer from various infectious diseases, which also include fungal infections (88–91). This is due to the weakened immune system of the patients, as well as the treatment used to cure COVID-19 infection. A study from 2020 showed that 91.8% of COVID-19 patients were reported to suffer from secondary infections caused by bacterial agents, while 23.3% suffered from fungal co-infections (92). Coronavirus-associated *Candida* infection (CACa) is the third most prevalent fungal disease associated with SARS-CoV-2, with a high mortality rate of 67.849% (91). However, it is necessary to state that the majority of these patients also had other complications or diseases, such as diabetes mellitus, hypertension, or obesity (93). Therefore, due to lack of documentation, it is unclear whether *C. auris* infection alone was responsible for the higher mortality rate or other risk factors significantly contributed toward it. Moreover, a recent meta-analysis showed that men are more frequently affected by CACa as compared to women, with a 3.7 times greater risk of co-infection. Another important fact is that COVID-19 infections may significantly alter host immune response, as a result of which a fungal pathogen can resist its disposal with usual antifungal drugs (94). However, there may also be the possibility that it is a consequence of drug-drug interactions, which are administrated against the COVID-19 and co-infections.

Overall, this study presents a refined strategy to improve the current computer-aided vaccine design. The major limitation of our study is the current lack of the experimental validation, including functional analysis to reveal if our epitopes can really evoke the immune responses in the cell lines and in appropriate animal models. This process is though very challenging, however, we hope that with the right research collaboration, we can switch our work from theoretical study to the experimental work. Our results are very promising despite getting only two epitopes from our conservative approach. Furthermore, we achieved good immunogenicity, antigenicity, and high populations coverage in our predicted vaccine candidates with least possibility of epitopes to get evolved or mutated.

## Conclusion

Vaccine design against invasive fungal pathogens has been challenging (95). In recent years, epitope-based vaccines have proven to provoke a potent and efficient defense response of the immune system in a targeted manner. They are synthesized at a lower cost, have good stability and relative safety, and have no limitations in target diseases. Thus, robust computational methods that have considerable predictive and analytical information combined with reverse vaccinology can encourage the prediction of novel epitopes that can serve as powerful vaccine candidates. Here, combining evolutionary information and employing an immune-informatics approach, we identified two potent vaccine candidates for designing a *C. auris* vaccine. This strategy provides a new approach to identify highly conserved putative T-cell epitopes for other emerging pathogens. We screened the whole *C. auris* genome and rejected the genes showing adaptive evolution. Using the highly conserved regions from candidate proteins, we further enabled the identification of two MHC class-II epitopes QTTCFQTEYYDPYIS and FVDPKKCCCDPKMIK, which besides showing high strain level conservancy, showed good immunogenicity, antigenicity, and no significant similarity with humans. In the follow up study, the results need to be experimentally confirmed by the peptide vaccine formulation in the laboratory followed by clinical trials.

## Data availability statement

The datasets presented in this study can be found in online repositories. The names of the repository/repositories and accession number(s) can be found in the article/Supplementary material.

## Author contributions

SG and MS designed the project and had the main conceptual ideas and made the first manuscript draft. ÖO, RM, SB, and EB performed the data analysis. SG, ÖO, and MS wrote the manuscript with feedback and the manuscript edits from all authors. TD provided the critical feedback, supervision, and helped to shape the research. All authors contributed to the article and approved the submitted version.

## Footnotes

1
http://imed.med.ucm.es/PVS

2
http://imed.med.ucm.es/Tools/antigenic.pl
