# Supplementary material for: Genome-wide scan for potential CD4+ T-cell vaccine candidates in Candida auris by exploiting reverse vaccinology and evolutionary information
Source: Front Med (Lausanne). 2022 Nov 3;9:1008527. doi: 10.3389/fmed.2022.1008527 (PMC9669072; doi:10.3389/fmed.2022.1008527)
Supplement: Supplementary file 1 [file Data_Sheet_1.PDF]

Supplementary Material

Genome-wide scan for potential CD4+ T-cell vaccine candidates in Candida auris by exploiting reverse vaccinology and evolutionary information

This PDF file includes:

1. Supplementary tables
   - Tables S1 – S3
2. Supplementary dataset
   - Dataset S1
3. Supplementary figures
   - Figures S1 – S4

* Corresponding author

Address correspondence to Prof. Dr. Thomas Dandekar, dandekar@biozentrum.uni-wuerzburg.de

	C. auris 6684	*C. auris* B8441	*C. auris* B11221	*C. auris* B11220	*C. auris* B11243
**Geographic location**	India	Pakistan	South Africa	Japan	Venezuela
**GenBank accession**	GCA_001189475.1	GCA_002759435.2	GCA_002775015.1	GCA_003013715.1	GCA_003014415.1
**Size (Mb)**	124,988	12,366	127,412	121,119	123,546
**GC%**	45,2	45,2	45,3	45	45
**Scaffolds**	99	15	20	320	238
**Scaffolds N50**	267017	1083522	2363944	60224	86716
**Contigs**	759	18	23	327	282
**Contigs N50**	30547	1033842	1467420	57519	77742

Table S1. Version and details of the *Candida auris* genomic assembly.

Table S2. List of immunogenic MHC-II binders screened from pipeline

SN	MHC-II binders
1	NAKNYWKEAICAIRA
2	GAGFAYFKVIDQLGQ
3	QTTCFQTEYYDPYIS
4	NCEHTPWWWTCFFEE
5	ANVGDMFDFLAIGGA
6	FVDPKKCCCDPKMIK

Table S3: Top 5 protein hits for epitopes covering all 5 clades. All hits have 100% identity and coverage with the epitopes.

**epitope1**						
Description	Total Score	E value	Acc. Len	Accession	Location	Clade
uncharacterized protein CJI97_001670	55.8	1.00E-11	296	XP_028892302.1	South Africa	III
hypothetical protein CJJ07_000063	55.8	1.00E-11	296	PSK80000.1	Venezuela	IV
hypothetical protein FDK38_000106	55.8	1.00E-11	296	QRG35783.1	Iran: Sari	V
hypothetical protein QG37_05332	55.8	1.00E-11	296	KND97917.2	India: Bengaluru	I
hypothetical protein CAJCM15448_29580	55.8	1.00E-11	295	GBL50684.1	Japan:Tokyo	II
**epitope2**						
Description	Total Score	E value	Acc. Len	Accession	Location	Clade
uncharacterized protein CJI97_002787	56.2	7.00E-12	527	XP_028890042.1	South Africa	III
hypothetical_protein	56.2	7.00E-12	522	QEO19793.1	Japan	II
hypothetical protein FDK38_000577	56.2	7.00E-12	507	QRG36243.1	Iran: Sari	V
hypothetical protein CJJ07_005539	56.2	7.00E-12	507	PSK74712.1	Venezuela	IV
hypothetical protein QG37_03918	56.2	7.00E-12	497	KND99125.2	India: Bengaluru	I

Dataset S1. Clusters of orthologues identified by OrthoFinder. Only the clusters containing annotated extracellular (red) or cell-membrane (blue) proteins are shown. Orthgroups in which positive selection signal was identified is marked with yellow highlight.

OG0000033: Caur6684|KND99350.2 Caur6684|KNE01154.1 CaurB11220|PSK48184.1 CaurB11220|PSK52343.1 CaurB11221|PIS50283.1 CaurB11221|PIS53642.1 CaurB11243|PSK76124.1 CaurB11243|PSK79256.1 CaurB8441|PIS49454.1 CaurB8441|PIS51654.1

OG0000194: Caur6684|KNE01476.2 CaurB11220|PSK51404.1 CaurB11221|PIS57664.1 CaurB11243|PSK75680.1 CaurB8441|PIS58219.1

OG0000942: Caur6684|KNE00198.2 CaurB11220|PSK45893.1 CaurB11221|PIS57792.1 CaurB11243|PSK75195.1 CaurB8441|PIS58330.1

OG0001068: Caur6684|KNE02721.1 CaurB11220|PSK51196.1 CaurB11221|PIS54408.1 CaurB11243|PSK79292.1 CaurB8441|PIS52296.1

OG0001725: Caur6684|KND99125.2 CaurB11220|PSK42774.1 CaurB11221|PIS53126.1 CaurB11243|PSK74712.1 CaurB8441|PIS51160.1

OG0002512: Caur6684|KND98132.2 CaurB11220|PSK49187.1 CaurB11221|PIS53395.1 CaurB11243|PSK76542.1 CaurB8441|PIS51411.1

OG0002621: Caur6684|KND98037.2 CaurB11220|PSK43872.1 CaurB11221|PIS53514.1 CaurB11243|PSK75506.1 CaurB8441|PIS51529.1

OG0002878: Caur6684|KND97382.2 CaurB11220|PSK46925.1 CaurB11221|PIS57033.1 CaurB11243|PSK77272.1 CaurB8441|PIS58604.1

OG0003103: Caur6684|KND97746.2 CaurB11220|PSK50589.1 CaurB11221|PIS55354.1 CaurB11243|PSK77748.1 CaurB8441|PIS54728.1

OG0003232: Caur6684|KND97160.1 CaurB11220|PSK38432.1 CaurB11221|PIS55948.1 CaurB11243|PSK78342.1 CaurB8441|PIS56872.1

OG0003238: Caur6684|KND97170.2 CaurB11220|PSK49031.1 CaurB11221|PIS55940.1 CaurB11243|PSK77354.1 CaurB8441|PIS56880.1

OG0003249: Caur6684|KND97185.2 CaurB11220|PSK49256.1 CaurB11221|PIS55928.1 CaurB11243|PSK75245.1 CaurB8441|PIS56891.1

OG0003318: Caur6684|KND97020.2 CaurB11220|PSK41673.1 CaurB11221|PIS55097.1 CaurB11243|PSK75240.1 CaurB8441|PIS54473.1

OG0003325: Caur6684|KND97034.1 CaurB11220|PSK38458.1 CaurB11221|PIS55105.1 CaurB11243|PSK75232.1 CaurB8441|PIS54481.1

OG0003693: Caur6684|KND96493.2 CaurB11220|PSK48779.1 CaurB11221|PIS53013.1 CaurB11243|PSK76241.1 CaurB8441|PIS51046.1

OG0003768: Caur6684|KND96266.1 CaurB11220|PSK49640.1 CaurB11221|PIS50122.1 CaurB11243|PSK78658.1 CaurB8441|PIS49616.1

OG0003916: Caur6684|KNE02050.2 CaurB11220|PSK44914.1 CaurB11221|PIS54234.1 CaurB11243|PSK76376.1 CaurB8441|PIS52249.1

OG0004120: Caur6684|KND95861.1 CaurB11220|PSK44728.1 CaurB11221|PIS54286.1 CaurB11243|PSK78133.1 CaurB8441|PIS52421.1

OG0004162: Caur6684|KND95788.1 CaurB11220|PSK43936.1 CaurB11221|PIS56026.1 CaurB11243|PSK75459.1 CaurB8441|PIS56794.1

OG0000005: Caur6684|KND98979.2 Caur6684|KND99597.2 CaurB11220|PSK35437.1 CaurB11221|PIS48192.1 CaurB11221|PIS49873.1 CaurB11221|PIS50647.1 CaurB11221|PIS50648.1 CaurB11221|PIS56524.1 CaurB11243|PSK74847.1 CaurB11243|PSK76051.1 CaurB11243|PSK76857.1 CaurB11243|PSK76858.1 CaurB11243|PSK79346.1 CaurB11243|PSK79348.1 CaurB8441|PIS49865.1 CaurB8441|PIS50296.1 CaurB8441|PIS50297.1 CaurB8441|PIS52481.1 CaurB8441|PIS52483.1 CaurB8441|PIS55432.1

OG0000411: Caur6684|KNE01031.1 CaurB11220|PSK47085.1 CaurB11221|PIS53727.1 CaurB11243|PSK79170.1 CaurB8441|PIS51739.1

OG0001407: Caur6684|KND99925.2 CaurB11220|PSK38785.1 CaurB11221|PIS57459.1 CaurB11243|PSK75210.1 CaurB8441|PIS59035.1

OG0001673: Caur6684|KND99280.1 CaurB11220|PSK47449.1 CaurB11221|PIS50231.1 CaurB11243|PSK75154.1 CaurB8441|PIS49507.1

OG0001714: Caur6684|KND99336.2 CaurB11220|PSK52354.1 CaurB11221|PIS50273.1 CaurB11243|PSK76135.1 CaurB8441|PIS49465.1

OG0001722: Caur6684|KND99351.2 CaurB11220|PSK52342.1 CaurB11221|PIS50284.1 CaurB11243|PSK76123.1 CaurB8441|PIS49453.1

OG0002125: Caur6684|KND98529.2 CaurB11220|PSK44034.1 CaurB11221|PIS50975.1 CaurB11243|PSK79720.1 CaurB8441|PIS50623.1

OG0002275: Caur6684|KND98787.1 CaurB11220|PSK49471.1 CaurB11221|PIS50805.1 CaurB11243|PSK78060.1 CaurB8441|PIS50454.1

OG0002576: Caur6684|KND97977.2 CaurB11220|PSK35317.1 CaurB11221|PIS53466.1 CaurB11243|PSK74667.1 CaurB8441|PIS51481.1

OG0002667: Caur6684|KND97917.2 CaurB11220|PSK51463.1 CaurB11221|PIS56420.1 CaurB11243|PSK80000.1 CaurB8441|PIS55538.1

OG0002882: Caur6684|KND97387.2 CaurB11220|PSK46929.1 CaurB11221|PIS57037.1 CaurB11243|PSK77268.1 CaurB8441|PIS58608.1

OG0003151: Caur6684|KND97823.1 CaurB11220|PSK46776.1 CaurB11221|PIS55406.1 CaurB11243|PSK77094.1 CaurB8441|PIS54780.1

OG0003223: Caur6684|KND97148.2 CaurB11220|PSK42760.1 CaurB11221|PIS55957.1 CaurB11243|PSK78351.1 CaurB8441|PIS56863.1

OG0003420: Caur6684|KND96900.1 CaurB11220|PSK47927.1 CaurB11221|PIS49381.1 CaurB11243|PSK77191.1 CaurB8441|PIS48769.1

OG0003714: Caur6684|KND96388.2 CaurB11220|PSK49561.1 CaurB11221|PIS52989.1 CaurB11243|PSK76660.1 CaurB8441|PIS51022.1

OG0004083: Caur6684|KND95951.2 CaurB11220|PSK47394.1 CaurB11221|PIS57098.1 CaurB11243|PSK79668.1 CaurB8441|PIS58670.1

OG0004084: Caur6684|KND95953.2 CaurB11220|PSK47393.1 CaurB11221|PIS57099.1 CaurB11243|PSK79669.1 CaurB8441|PIS58671.1

OG0004411: Caur6684|KND95458.1 CaurB11220|PSK39528.1 CaurB11221|PIS53927.1 CaurB11243|PSK76575.1 CaurB8441|PIS51941.1

OG0004712: Caur6684|KNE01560.1 CaurB11220|PSK41708.1 CaurB11221|PIS49106.1 CaurB11243|PSK79900.1 CaurB8441|PIS48493.1

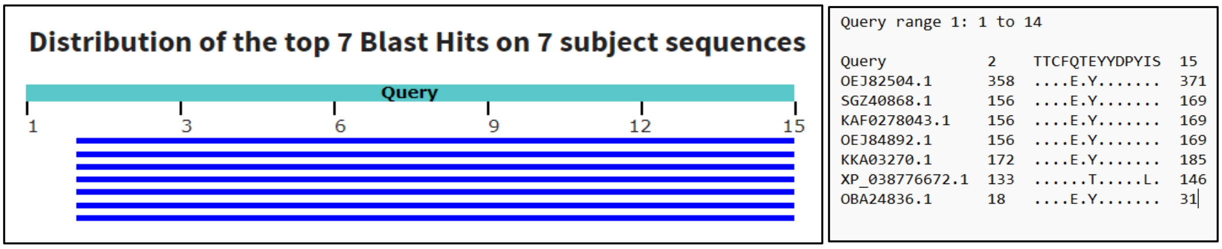

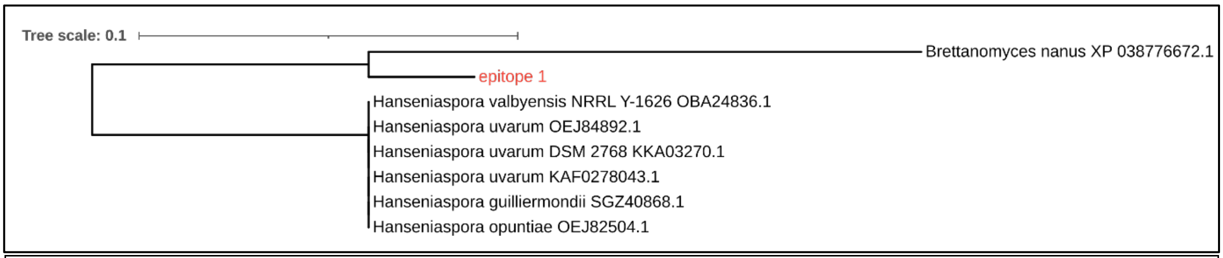

Description	Scientific Name	Total Score	Query Cover	E value	Per. ident	Acc. Len	Accession
Cell surface superoxide dismutase [Cu-Zn] 6	Hanseniaspora opuntiae	42.6	93%	4E-04	85.71	550	OEJ82504.1
uncharacterized protein HGUI_03068	Hanseniaspora guilliermondii	42.6	93%	4E-04	85.71	350	SGZ40868.1
hypothetical protein FOG50_01120	Hanseniaspora uvarum	42.6	93%	4E-04	85.71	348	KAF0278043.1
Cell surface superoxide dismutase [Cu-Zn] 6	Hanseniaspora uvarum	42.6	93%	4E-04	85.71	348	OEJ84892.1
hypothetical protein D499_0C02580	Hanseniaspora uvarum DSM 2768	42.6	93%	4E-04	85.71	271	KKA03270.1
uncharacterized protein FOA43_000412	Brettanomyces nanus	42.6	93%	4E-04	85.71	257	XP_038776672.1
hypothetical protein HANVADRAFT_50585	Hanseniaspora valbyensis NRRL Y-1626	42.6	93%	4E-04	85.71	215	OBA24836.1

Figure S1: Top 7 hits obtained from the blast search against fungal proteins for identified epitopes (e-value < 0.01). Only epitope 1 was found to have similarities with proteins from Hanseniaspora species and Brettanomyces nanus.

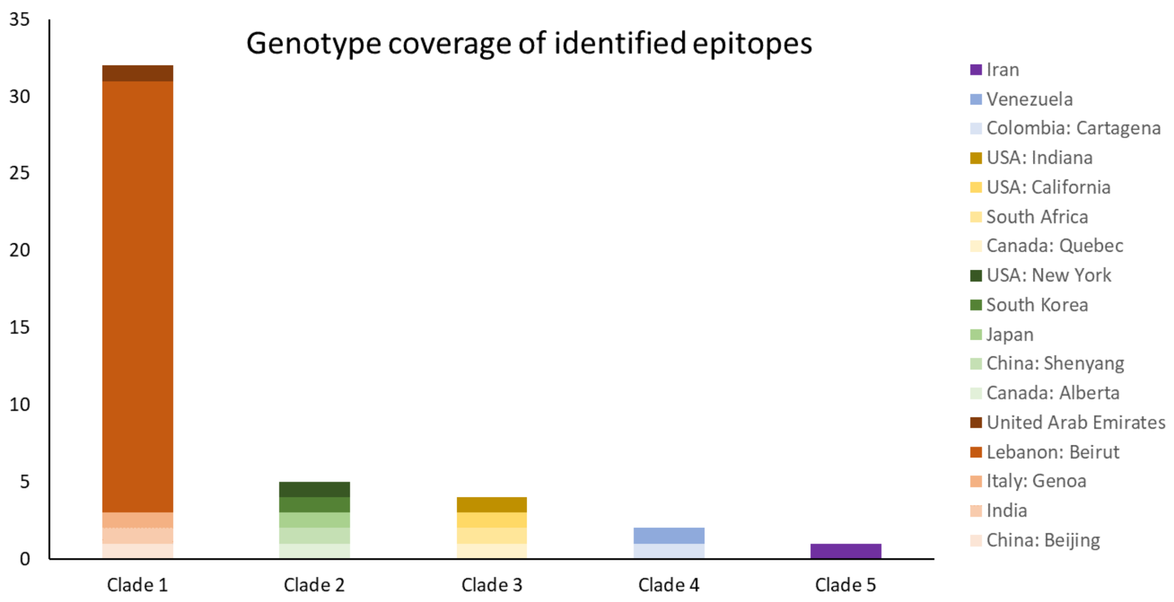

Figure S2: Both epitopes have homologs spanning all 5 C.auris clades and 17 strains.

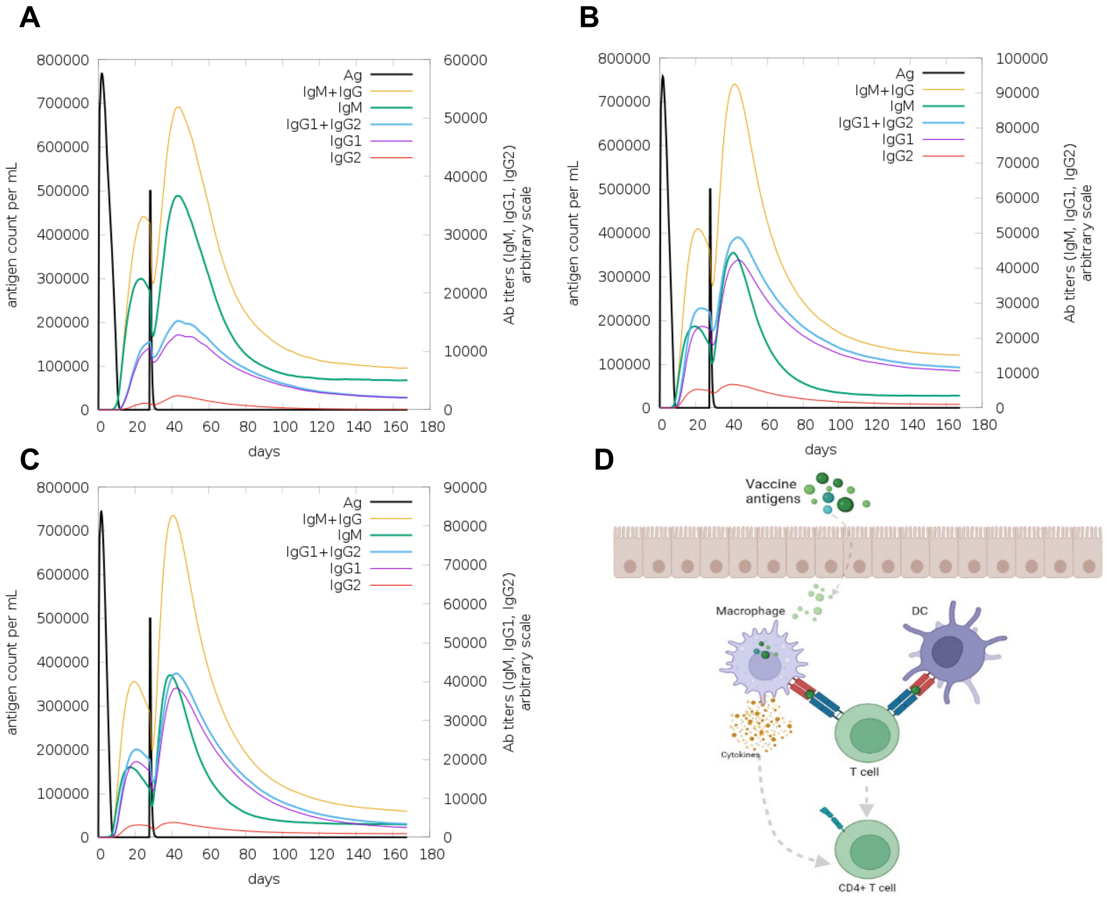

Figure S3: Antigen count and the immunoglobulins and the immunocomplexes in response to epitopes. Injections given on day 1 and after 4 weeks. 3 simulations performed against different HLA types: (A) HLA-DRB1*13:01 and HLA-DRB1*03:01 (B) HLA-DRB1*13:01 and HLA-DRB3*01:01 (C) HLA-DRB1*03:01 and HLA-DRB3*01:01. (D) Figure depicts the mechanism of the vaccine. The peptide-based vaccine is injected into the body, the antigen is recognized by the immune cells, which trigger specific B- and T-cells response. The T-cells recognize the peptide sequences complexed with MHC class II molecules. CD4+ T-cells recognize MHCII-epitope complexes and interacting with cytotoxic T-cells, which lead to the neutralization the pathogen.

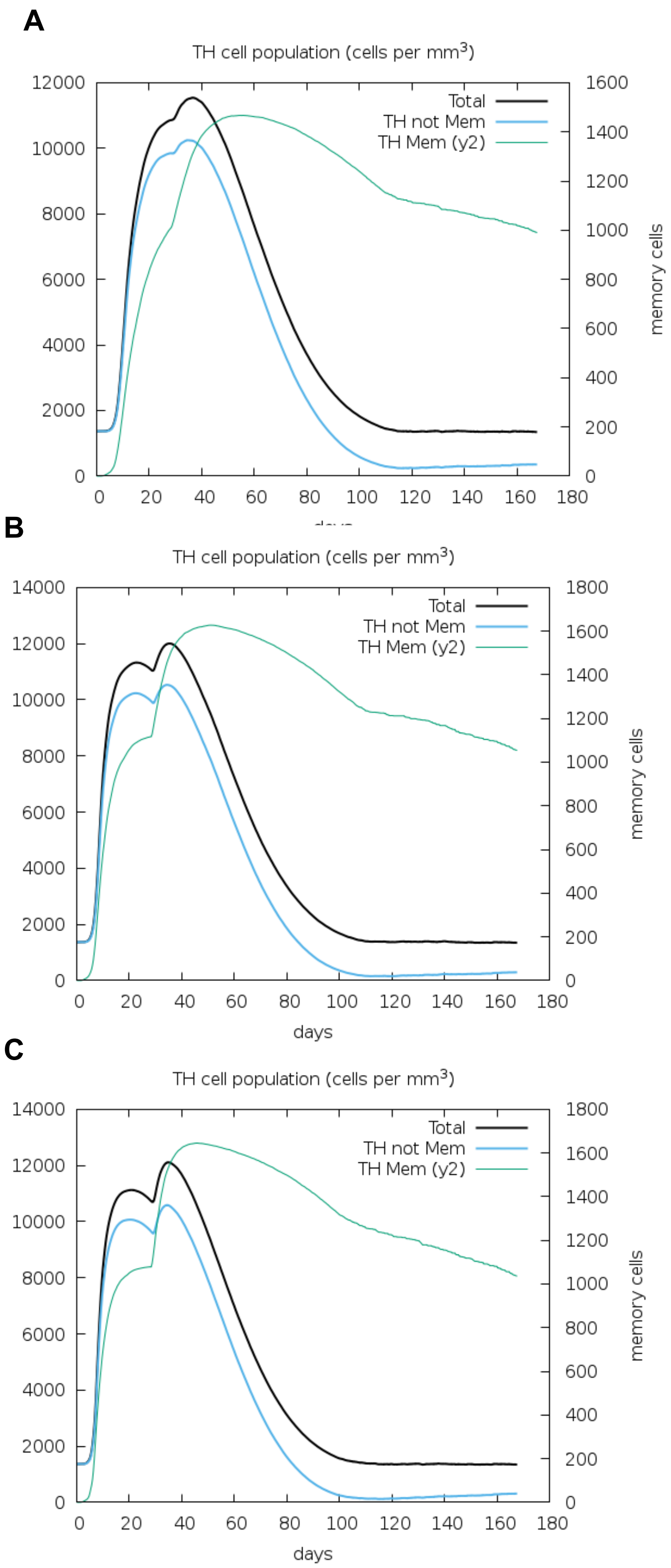

Figure S4: CD4 T-helper lymphocytes total and memory counts in response to epitopes. Injections given on day 1 and after 4 weeks. 3 simulations performed against different HLA types: (A) HLA-DRB1*13:01 and HLA-DRB1*03:01 (B) HLA-DRB1*13:01 and HLA-DRB3*01:01 (C) HLA-DRB1*03:01 and HLA-DRB3*01:01.
